# Research Progress on the Physicochemical Properties of Starch-Based Foods by Extrusion Processing

**DOI:** 10.3390/foods13223677

**Published:** 2024-11-19

**Authors:** Chao Qiu, Han Hu, Baicun Chen, Qianzhu Lin, Hangyan Ji, Zhengyu Jin

**Affiliations:** State Key Laboratory of Food Science and Resources, School of Food Science and Technology, Collaborative Innovation Center of Food Safety and Quality Control in Jiangsu Province, Jiangnan University, Wuxi 214122, China; phdqiu@jiangnan.edu.cn (C.Q.); 6230112023@stu.jiangnan.edu.cn (H.H.); 6230111171@stu.jiangnan.edu.cn (B.C.); lqz20172208008@163.com (Q.L.); jihangyan@jiangnan.edu.cn (H.J.)

**Keywords:** extrusion, starch, physicochemical properties, food composition, functional food

## Abstract

Extrusion is a crucial food processing technique that involves mixing, heating, shearing, molding, and other operations to modify the structures and properties of food components. As the primary energy source material, the extrusion process induces significant physical and chemical changes in starch that impact the quality of final products. This review paper discusses novel technologies for starch extrusion and their influence on the physical and chemical properties of starch-based foods, such as gelatinization and retrogradation properties, structural characteristics, and digestion properties. Additionally, it examines the application of extrusion in starch processing and the interactions between starch and other food components during extrusion. This information sheds light on the structural and property alterations that occur during the extrusion process to create high-quality starch-based foods.

## 1. Introduction

Extrusion processing technology is an advanced method that integrates mixing, stirring, crushing, heating, steaming, sterilization, puffing, and shaping into a single process. Extrusion technology offers numerous advantages over other food processing techniques, such as minimal nutrient degrassssdation, reduced production time, lower risk of microbial contamination, product diversity, strong adaptability to various raw materials, low energy consumption, simplicity of operation, and high production efficiency. Its significant practical value has led to its widespread application in both the food and feed industries [1]. Food extrusion processing involves forming products with specific shapes and structural characteristics by subjecting the material to intense mechanical forces within a specially designed die after it undergoes pretreatment processes, including grinding, humidifying, and mixing.

Starch is a major source of human energy and is widely found in seeds, roots, tubers, stems, leaves, and fruits. However, most natural starches’ properties have many inherent limitations that limit their application, to some extent, in various fields [2]. Mitigating these limitations has always been the main object of research into the processing of grain-based foods by extrusion [3], and the quality of the final product is largely determined by the structural and chemical changes of starch during processing [4]. During extrusion, the high temperature, high pressure, and high shear forces in the extruder chamber can cause structural reorganization and complex physicochemical changes, such as gelatinization and degradation, in starch [5,6]. Therefore, extrusion processing technology can often impart more desirable properties to starch and better adapt to the diverse needs of consumers for starch-based foods. In recent years, as a profound understanding and effective control of the extrusion process have been gained, extrusion modification technology has been increasingly applied in starch food processing. As a multifunctional reactor, the extruder can study and develop various components with enhanced functions through extrusion, increase the interaction between starch and these components, and then adjust the characteristics of starch recovery, digestion, and so on, enabling its better use in food processing [7]. This process ensures the proper dispersion of these embedded components while inhibiting starch resurgence to achieve the controlled and slow release of properties [8]. High moisture extrusion has demonstrated significant retention of total phenolic compounds and flavonoids, along with the highest content of slow-digested starch [9]. Furthermore, researchers have successfully employed the synergistic effect between amylase degradation and extrusion to enhance protein digestibility in non-glucosidic chia seed flour [10], alongside utilizing extrusion-based 3D printing processes to meet the increasing demand for personalized and customized foods within the food industry [11].

The objective of this review is to provide an overview of the application of extrusion processing technology in starch and its effects on particle structure, gelatinization, and texture properties. The change of structure and function in single-component starch during the extrusion process cannot be ignored in considerations of food quality performance. However, the interaction of starch with other components is also important for understanding the changes in quality of starch-based foods and the design of high-quality products. Therefore, this paper summarizes the interactions between starch and protein, fats, polysaccharides, and polyphenolic substances during extrusion to provide general insights into starch-based food extrusion and guide product design and process control.

## 2. The Latest Extrusion Processing Technology

### 2.1. High-Moisture Extrusion

In the extrusion process, moisture content plays a crucial role and is often considered a key parameter. The material itself contains varying amounts of water, and water or steam can also be added directly during mixing and feeding. As a plasticizer, water actively participates in many reactions. Therefore, water softens the resulting substance, changes its rheology, and promotes starch gelatinization [12,13]. Starch gelatinization depends on moisture content, temperature, shear force, and time. It is worth noting that the moisture content can characterize the process itself. When the humidity in the extrusion process is low, that is, when the moisture content is small, the degree of gelatinization of the starch will be limited. The extrusion process under low moisture conditions is often referred to as “dry extrusion”. In the process of dry extrusion, due to the lack of water, starch particles have difficulty fully absorbing water and expand and break, resulting in a low gelatinization degree. This can affect the taste and texture of starchy foods, making them hard and dry [14]. On the contrary, when the humidity in the extrusion process is higher, that is, when the moisture content is higher, the gelatinization degree of starch is significantly improved. High-moisture extrusion, also known as “wet extrusion”, promotes the gelatinization of starch by increasing the moisture content (>40%), making it easier for starch particles to absorb water and expand and break up [15]. This gelatinization process not only improves the taste and texture of starchy foods but also makes them easier to digest and absorb [16]. In addition, low-water extrusion technology is carried out under the conditions of low water and high temperature, the energy consumption is large, the shear effect is strong, the wear of the barrel and screw is large, and the destruction of nutrients in the material is also serious [17]. High-moisture extrusion can be carried out at low temperatures and the cost is lower.

Aasima et al. [9] used a twin-screw extrusion mechanism to produce pre-gelatinized pasta and proved that the degree of gelatinization was positively correlated with temperature and feed moisture. Another study further showed that high-moisture extrusion could improve the retention rate of spices such as isovalerate, ethyl butyrate, and butyrate in granular extrusions [18]. In addition, the high water content in the extrusion process increases the proportion of slow-digested starch, thus affecting the digestibility of starch [19]. Kim et al. [20] studied the effects of different feed moisture levels (20%, 40%, and 60%) on the formation of resistant starch (RS) in pastry flour and observed a very significant positive correlation between moisture and RS formation compared with unextruded pastry flour samples.

### 2.2. Enzymatic Extrusion

Enzyme extrusion is an emerging extrusion technology that involves the addition of exogenous enzyme preparations during the extrusion of materials [21]. The extruder functions as a special enzyme reactor to enhance the modification or degradation of biological macromolecules, such as starch, protein, and cellulose. Various enzymes, including amylase, protease, cellulase, and lipase, have been introduced into the extrusion process to improve substrate conversion [22]. During enzyme-assisted extrusion, the extruder chamber provides a substrate-rich environment for efficient enzymatic reactions. This “enzyme package center radiates to the surrounding” structure, accelerates reaction coordination, improves the substitution rate between the enzyme and the substrate active center, and significantly improves the overall efficiency of the enzyme [23,24]. Vanier et al. [25] studied natural rice, legumes, and corn starch with different ratios of straight chain and branched chain and found that the sample containing 8% amylose had lower elasticity, hardness, and viscosity and formed a thinner wall layer of porous bubbles. It is worth noting that several factors during the extrusion process can significantly affect the behavior of the enzyme. First of all, temperature is a crucial factor that can not only affect the activity of enzymes but also change the gelatinization characteristics of starch, thus affecting the interaction between enzymes and starch [24]. Secondly, the change in extrusion speed also has a significant effect on the behavior of the enzyme. Increasing the rotation speed of the screw can increase the shear strength, thus destroying the hydrogen bond between starch chain molecules, accelerating the gelatinization process and making starch particles more likely to expand and crack, thus providing more accessible molecular centers for enzymes and promoting the enzymatic hydrolysis reaction [1,25]. In addition, water content is also a factor that cannot be ignored and can not only affect the gelatinization degree of starch but also affect the diffusion and catalytic efficiency of enzymes [18].

Enbo et al. [26] utilized enzymatically extruded rice as an adjunct material for rice wine fermentation, which effectively retained non-substrate active compounds, shortened the fermentation time, and improved the quality of the rice wine. Furthermore, enzymatic extrusion technology has made significant progress in preparing porous starch-based materials. Li et al. [27] employed a medium-temperature α-starch enzyme to extrude wheat flour with enzymes to produce extruded instant noodles with a porous structure. Compared to traditional noodles, the multi-porous structure endowed the extruded noodles with an excellent water absorption ability. Xu et al. [28], on the other hand, used metal ions and high-temperature-resistant α-amylase, respectively, to activate starch during the enzymatic extrusion process, promoting the enzyme-mediated degradation of starch and facilitating the formation of a desirable porous structure during extrusion.

### 2.3. Hot-Extrusion 3D Printing

Hot-extrusion three-dimensional (3D) printing technology (HE-3DP) involves heating raw food materials in a cylinder and extruding them through an extrusion nozzle, allowing the extruded materials to solidify or maintain a specific fluidity and viscosity for a certain period [29]. By moving nozzles and platforms in 3D space, a layer-upon-layer deposition is achieved, enabling the manufacturing of 3D food [11]. As an innovative physical processing technology, HE-3DP has intelligent, personalized, green, and environmentally friendly characteristics. It caters to diverse individuals’ pursuit of food quality, nutritional functionality, and fashionable designs while providing a novel approach to realizing the personalized and precise customization of nutritious and healthy food [30].

The rheological properties of starch contribute to its excellent formability in HE-3DP, and the combined effects of shear force, heat energy, and water molecules during the process induce the evolution of starch’s multi-scale structure, leading to changes in its digestive properties and nutritional functions [31]. Lille et al. [32] developed a paste system consisting of protein, starch, and fiber-rich materials for 3D printing. Their research demonstrates that the rheological properties of printed ingredients significantly impact formability, requiring not only high structural strength but also a certain level of deformation resistance to ensure stable shape retention after printing. Godoi et al. [33] utilized soft materials, such as dough and minces, as extrusion 3D printing materials; their results reveal that the printability of soft materials is greatly influenced by their inherent rheological properties. At high shear rates, soft materials should possess low enough viscosity for smooth extrusion from the nozzle while quickly recovering to a higher viscosity post-extrusion to meet adhesion requirements between sedimentary layers. Fanli et al. [34] examined how potato starch influences both rheological and mechanical properties within lemon juice gel systems, affecting the structural accuracy and shape stability in printed products. Their study ultimately led to the development of a novel type of 3D-printed food material utilizing a mixed gel system comprising lemon juice and potato starch.

### 2.4. Improved Extrusion Cooking Technology (IECT)

The traditional extrusion cooking technology is a well-established method in the history of food processing, characterized by high temperatures and short processing times. During this process, wet-expandable starches undergo significant physical transformations [35]. In contrast to the conventional single-screw extrusion technique, IECT represents a novel form of extrusion gelatinization technology. The improved single-screw extruder exhibits distinctive features, such as an extended screw length (1950 cm), longer residence time (18–90 °C), a lower temperature range (50–150 °C), and reduced screw speed (15–75 rpm). These differences result in material expansion and structural modifications of the extrudate, thereby enhancing its properties [36].

The potential application of IECT technology is expected to revolutionize the physical and chemical properties of starch, thereby imparting desirable functional properties to food. Research has demonstrated that rice prepared using IECT exhibits high nutritional value and retains similar texture characteristics and shape as traditional rice [36]. Furthermore, modified high amylose starch prepared through IECT shows a reduced degradation rate and improved stability [37]. Additionally, this technology enhances the freeze–thaw stability of starch [38].

## 3. Effect of Extrusion Processing Technology on the Structure and Physicochemical Properties of Starch

Starch undergoes a transition between ordered and disordered structure during the extrusion process, during which starch particles rapidly absorb water and expand, leading to starch gelatinization [39]. In addition, after extrusion, melted starch molecules can rejoin into a double helix during cooling, leading to starch retrogradation [40]. 

### 3.1. Molecular Structure of Starch

Extrusion can have a significant impact on the molecular weight distribution, leading to substantial degradation of starch polymers [41]. The molecular weight of starch polymers greatly influences the physical properties of starch, such as solubility and viscosity [42]. Therefore, it is crucial to modify the molecular structure of natural starch through extrusion in order to obtain starch and its derivatives that meet specific requirements and expand the application range, thereby establishing a processing–structure–performance relationship [43].

The results demonstrate that extrusion plays a dominant role in reducing the size and crystallinity of starch molecules by preferentially breaking internal bonds within these molecules. Moreover, there is a significant change in the size distribution of amylopectin, while amylose remains relatively unaffected. Consequently, rigid microcrystals formed by amylopectin within starch particles are more susceptible to shear degradation during extrusion compared to flexible amorphous amylose [40]. Additionally, amylose also undergoes degradation during extrusion, resulting in shorter chains and a reduced iodine-binding capacity [44]. Sarawong et al. [45] reported an increase in raw banana powder’s amylose content due to α-1,6 glucoside bond breakage within amylopectin caused by shearing forces exerted during extrusion.

### 3.2. Starch Gelatinization and Retrogradation

Extrusion involves thermal energy and mechanical energy, which may destroy the crystal structure of starch to a large extent and have certain effects on the texture, flow, and digestion of starch [40]., as shown in Table 1. The gelatinization of starch plays a crucial role in the processing of starch and starch-based foods. During the extrusion process, starch particles rapidly absorb water and swell, while heat, shear forces, and pressure induce starch gelatinization, resulting in a transition from an ordered to a disordered structure [46]. Starch gelatinization during extrusion occurs quickly under low-moisture conditions with minimal energy consumption. The variation in gelatinization characteristics primarily arises from the breakdown of the starch particle structure and amylose leaching [47]. Furthermore, the degree of starch gelatinization is influenced by extrusion parameters. Extrusion causes pre-gelatinization of starch, disruption of its particle structure, weakening of hydrogen bonds, and reduction in residual starch particles with increasing screw rotation speed. Consequently, viscosity decreases as rotation speed increases [48].

Ruihan et al. [60] demonstrated a significant enhancement in the water absorption index, water solubility index, swelling power, and starch gelatinization degree of quinoa flour following extrusion treatment compared to its pre-extrusion state. Liu et al. [61] investigated the impact of varying water content (30%–70%) on rice starch gelatinization after extrusion and observed a decrease in the peak viscosity with increasing water content, which was attributed to the expansion of starch particles. Starch gelatinization occurred during the extrusion process, leading to reduced residual granulated starch and a decreased swelling degree, resulting in a notable reduction in peak viscosity. Jiejie [49] examined changes in the physicochemical structure and in vitro digestibility of extruded products by incorporating purple sweet potato into rice and found that compared to pure rice-based extruded products, those with added purple sweet potato exhibited enhanced antioxidant properties and higher water absorption index and water solubility index values, as well as lower enthalpy (ΔH) values. These findings provide evidence that starch gelatinization requires less energy and results in lower relative crystallinity.

On the other hand, gelatinized starch molecules are reformed through hydrogen bonding between amylose and amylopectin molecules during the cooling process. This leads to the formation of cross-linking and local crystallization regions in the starch, resulting in retrogradation [50]. In the process of extrusion, starch particles are subjected to high shear force and high heat, their ordered structure is destroyed, the hydrogen bond between starch molecules is broken, and the gelatinization degree of starch particles is increased. This destruction makes it difficult for starch molecules to form tight structures when they are rearranged, thus delaying the aging process [51]. Sarawong et al. [52] discovered that extruding banana starch under low-moisture and high rotational speed conditions resulted in molecular degradation and a decrease in the starch recovery value. The findings indicate that extrusion could inhibit short-term retrogradation of banana starch. von Borries-Medrano et al. [62] found significant changes in the structure and properties of cornstarch samples after reacting them with galactomannan and lemon during extrusion treatment. This suggests that extrusion treatment has a notable impact on both starch retrogradation and resistant starch formation. Based on these properties, extruded starch can not only be added to instant food products but also maintain good taste for several hours after cooking while being suitable for preparing starchy foods with slow digestion characteristics.

### 3.3. Starch Rheology

Viscosity is an important parameter in the description of the rheological properties of starch because starch can be used as a thickener in different foods [53,54]. Extrusion has a significant impact on the viscosity of starch because the larger mechanical shear force during the extrusion process causes the internal glycoside bond of starch molecules to break, promotes the hydrolysis and degradation of starch, and reduces the viscosity of starch [55]. For example, the viscosity of rice flour starch decreases after extrusion [63]. This is due to the fact that extrusion makes the hot paste’s viscosity and the apparent viscosity of starch with a low amylose content lower than that of starch with a high amylose content [64]. The power-law model has a high fitting degree for the viscosity model of rice flour extrudates with low amylose content, while the Herschel–Bulkley model has a high fitting degree for the extrudates of rice flour with a high amylose content [65]. Therefore, low viscosity, especially low peak viscosity, is a characteristic of extruded starch.

Starch viscosity is a key index to evaluate the quality and application value of starch and is affected by many factors, including extrusion parameters, and many processing conditions will have a significant impact on starch viscosity. During the extrusion process, the extrusion temperature can be precisely controlled by the heating system so as to adjust the viscosity [17]. Secondly, increasing the screw speed can increase the shear strength, destroy the hydrogen bond between the starch chain molecules, and accelerate the gelatinization process. This causes the starch particles to expand and crack, exposing more starch molecules and thus increasing viscosity. However, too high a screw speed may also cause excessive degradation of starch and reduce viscosity [66]. In addition, an appropriate amount of water can increase the viscosity of the starch solution [15]. In the extrusion process, precise adjustment of the viscosity can be achieved by controlling the amount of water added [13].

The effect of extrusion on the viscosity of starch is one of the main physical and chemical properties that determines the application of starch and starchy food. For example, the important product property of starch instant powder is the viscosity of soluble gel [66]. The use of natural arrowroot powder is limited by its high gelatinization temperature and high viscosity, resulting in a poor consumer experience. The treatment of pueraria starch by extrusion swelling modification was studied. After modification, the gelatinization temperature of puerariae starch decreased (except in a urea alkaline treatment), and the apparent viscosity of puerariae starch decreased from 517.95 Pas to 0.47 Pas [67].

### 3.4. Starch Digestion

The most common result of food extrusion is the degradation of starch particles, which improves their digestibility. High shear stress during extrusion treatment will destroy the integrity of starch powder particles and increase the contact area between starch and amylase during hydrolysis [56]. Zhang et al. [68] studied the structural and functional property changes of starch-based polymers in flour during low-moisture extrusion processing. The Maillard reaction and caramelization reaction that occurred during extrusion led to a darkening of the powder color, and the degradation of starch molecules led to the formation of surface cracks, pits, and holes. Garcia-Valle et al. [69] found that mango and amaranth starch gelatinization occurred after extrusion treatment, which destroyed its ordered structure and increased the content of slow-digesting starch.

However, most of the current research reports are more focused on using extrusion modification to reduce the digestibility of starch [57]. Consequently, numerous scholars have embarked on studying the extrusion reaction of starch with other substances (such as fatty acids, citric acid, and galactomannan) in order to augment the content of resistant starch in extruded starch. These studies explore the interaction between components and investigate the effects of exogenous additives on starch structure and digestibility [58,59]. Jiangping et al. [70] employed a one-step reactive extrusion method to synthesize citrate-esterified rice starch, which resulted in enhanced cross-linking of starch and a significant increase in the resistant starch content (*p* < 0.05). Studies have reported that the formation of a starch–lipid complex can elevate the resistant starch content in starchy materials [71]. Cervantes-Ramirez et al. [72] investigated the functional properties of amylopectin lipid complexes formed during mixed extrusion processes involving cornstarch and fatty acids such as stearic acid, oleic acid, and corn oil. The results indicate high levels of amylose lipid complexes formed through extrusion with stearic acid. von Borries-Medrano et al. [62] examined different types of galactomannan additives (e.g., guar gum, tala gum, locust bean gum) along with citric acid during an extrusion treatment for enhancing recovery rates and promoting resistant starch formation in processed starches. The findings reveal an increased V-shaped structure when storing the extruded product at 4 °C after being treated with citric acid and various types of galactomannans.

## 4. Application of Extrusion Processing Technology in Starch

### 4.1. Resistant Starch

During the extrusion process, starch undergoes significant changes in structure and properties due to the effects of heat, shear force, and pressure. Extrusion can destroy the covalent hydrogen bond and crystal structure of starch, which is conducive to the rearrangement of starch molecules and promotes the formation of RS [73,74]. Liu et al. [75] investigated the impact of extrusion and recrystallization treatments on the structure, physicochemical properties, and digestibility of corn starch and potato starch. The findings reveal that the extrusion treatment resulted in a decrease in molecular weight compared to natural starch while increasing the apparent amylose content. The RS content in corn starch treated with extrusion and recrystallization was approximately 6.5 times higher than that of raw corn starch (2.02%).

He et al. [76] prepared a V-type complex of rice starch and guar gum through extrusion treatment and analyzed its structure and digestibility. The results demonstrate that the complex exhibited a higher RS content with a more compact structure than rice starch alone. These findings suggest that guar gum could partially inhibit starch retrogradation. Feng et al.’s [77] study indicates that when starch is bound to negative polysaccharides, such as sodium alginate or xanthan gum, there is a tighter interaction between these polysaccharides. Thus, a greater steric hindrance is formed for digestive enzymes binding to starch than in positive polysaccharides, such as chitosan, which renders starch less resistant to digestion. Cui et al. [78] employed HE-3DP technology to fabricate a wheat starch–caffeic acid complex. The findings reveal that the application of high shear force and hydrothermal treatment led to the disruption of the crystal structure of wheat starch. The caffeic acid formed a highly organized B + V crystalline structure through hydrogen bonding with wheat starch, exerting competitive and mixed inhibitory effects on α-amylase and α-glucosidase activities, respectively. In vitro digestion experiments demonstrated that an increase in caffeic acid content significantly reduced rapidly digestible starch (RDS) while slowly increasing the digestible starch (SDS) and RS content, as well as decreasing the digestibility of wheat starch. These results provide valuable insights for the development and formulation of starchy foods with a low glycemic index (GI) using HE-3DP technology.

At present, extrusion technology is widely used in the field of RS (resistant starch) by adjusting a series of extrusion parameters, which can significantly affect and modify the structure of natural starch, thereby transforming it into RS. Typically, starch is extruded under specific conditions to produce low-GI products, such as low-GI complex rice, instant meal powder, and protein bars. Saadat et al. [79] prepared protein bars by combining extruded flour with whey protein concentrate, honey, and palm oil, resulting in products with in vitro and in vivo digestibility ranges of 62.04% to 74.98% and 65.30% to 84.01%, respectively. Zhang et al. [65] studied two extrusion methods of instant food powder with Tartary buckwheat powder and red bean powder as the main components, aiming to explore the specific effects of different extrusion parameters on the plants’ chemical compositions, physicochemical properties, and in vitro starch digestibility. The results show that the A-glucosidase inhibitory activity (45.26%) of instant powder obtained by single extrusion (25% moisture) was higher than that obtained by mixed extrusion (58.39%), which further demonstrates the key role of extrusion parameters in regulating the conversion of starch to RS.

### 4.2. Pre-Gelatinized Starch

Pre-gelatinized starch is a physically modified starch commonly utilized as an auxiliary material to enhance the processing performance and sensory attributes of food, as it can form a cold-water paste and exhibits a high water absorption capacity [80]. Extrusion technology can be employed for the production of pre-gelatinized starch since it efficiently gelatinizes starch. In the extrusion process, pyrolysis refers to the chemical decomposition process in which organic matter (in this case, starch) is heated in an oxygen-free or anoxic state, breaking its polymer bonding state and making it into gaseous, liquid, or solid small molecules (such as soluble small molecules and dextrins). This process increases the water solubility of starch and is one of the key steps in the production of pre-gelatinized starch by extrusion technology [81]. Extrusion parameters have significant influence on the pre-gelatinization process, including temperature, pressure, and shear force. A high temperature is conducive to the gelatinization of starch molecules, while appropriate pressure and shear force can promote the rupture of starch particles and the formation of dextrin, thereby optimizing the performance of pre-gelatinized starch. Therefore, the process of producing pre-gelatinized starch by extrusion technology involves the application of heat in high temperature and short-term continuous cooking, the generation of pressure, and a strong shear force, which, together, act on the starch molecules to achieve an efficient gelatinization process through pyrolysis [82]. Martinez et al. [83] discovered that incorporating pre-gelatinized rice flour treated with high-intensity extrusion could yield gluten-free rice dough with an elevated elastic modulus and consistency.

Pre-gelatinized starch produced through extrusion technology is used in various convenience foods, such as nutritional pastes and instant soups, where it serves to thicken and enhance flavor. Extruded pre-gelatinized starch is incorporated into wheat flour to improve the smoothness and overall quality of noodles [84]. Research indicates that extruded pre-gelatinized flour can effectively substitute additives, such as pre-gelatinized starch and hydrophilic glue, addressing the lack of gluten in gluten-free flour that leads to weak dough formation during water kneading [85]. Pre-gelatinized starch significantly influences the texture properties and baking characteristics of dough. The addition of extruded wheat aleurone reduces dough formation time while increasing its stability period [71]. This is attributed to the degradation of starch molecules during the extrusion process, which breaks glucoside bonds and forms a porous structure with a high water absorption capacity in the resulting pre-gelatinized starch. Consequently, baked products exhibit increased volume along with improved moisture retention and desirable structural integrity [86]. Bread in which extruded pre-gelatinized starch is used has a smoother appearance with a more uniform surface coating structure than bread made from natural starch. Additionally, bread incorporating extruded wheat starch exhibits a larger specific volume. As the temperature used for extrusion increases, so does the extent of damage inflicted on the starch molecules, leading to an increase in specific volume for bread products [87].

### 4.3. Porous Starch

Extrusion treatment results in loss of integrity, disintegration of starch particles, and degradation of starch molecules. Therefore, when starch is passed through the extruder mold under pressure reduction and water evaporation, the melted extruder usually expands, causing the starch to form a porous honeycomb structure when it leaves the extruder, which is porous starch [1]. Schweiggertk et al. [88] showed that starch has cross-linking and other effects during extrusion. Therefore, mechanical extrusion is conducive to the formation of a porous starch structure, and the porosity of porous starch is positively correlated with temperature. The key parameters in the process of mechanical extrusion, such as temperature and screw speed, have a significant effect on the formation of porous starch structure [42]. In particular, temperature not only affects the gelatinization degree of starch but also directly affects the expansion degree of extrudates and the development of the pore structure. Generally, with the increase of temperature, the activity and plasticity of starch molecules are enhanced, which is conducive to the formation of a more open and uniform porous structure [7]. Therefore, the porosity of porous starch is often positively correlated with the temperature during extrusion. In addition, an increase in screw speed can produce a higher shear force, contributing to the further crushing and gelatinization of starch particles, which may promote the thinning and increase in the number of pores. However, too high a speed can also cause the extrudate to overheat and degrade, so it is necessary to find the best speed balance [6].

Enzymatic synergistic preparation of porous starch is widely employed due to its ease of use [31]. In enzymatic extrusion processes using metal ions and heat-resistant α-amylase for activating starch degradation, respectively, favorable porous structures are formed during extrusion [28]. Ho et al. [89] bioextruded starch using a twin-screw extruder, followed by hydrolysis with α-amylase at a moderate temperature to prepare porous starch with a specific surface area of 2.52 m^2^/g, a total pore volume of 4.53×10^−3^ cm^3^/g, and an average pore size of 7.36 nm. With an oil adsorption capacity of up to 63.29%, it has the advantages of high efficiency, strong substrate specificity, and strong environmental protection.

## 5. Effect of Extrusion Processing Technology on the Interaction of Starch-Based Food Components

Extrusion technology is emerging as an efficient processing method that can simultaneously induce shearing, heating, and pressure in a precisely controlled manner. This enables precise modifications to the structure of starch molecules and enhances interactions with other non-starch molecules, as demonstrated in Table 2.

### 5.1. Starch and Protein

In most cases, starch and protein are the main components of a food [95], and the way they interact largely determines the food’s nutritional and textural quality. The interaction between starch and protein is not a single force but includes covalent bonds, electrostatic interactions, hydrogen bonds, van der Waals forces, and hydrophobic interactions [96]. Extrusion promotes cross-linking and polymerization between protein and starch through the regulation of various factors to form polymers with improved nutritional and functional properties. Tellez-Morales et al. [97] conducted a single-screw extrusion experiment using a blend of corn aleurone and whey protein isolate as raw materials. The interaction of starch and protein during extrusion in the food system was simulated. During the extrusion process, protein hydrolyzes, denatured structures unfold and rearrange, and the sites of cross-linking between protein and starch increase with the increase of protein concentration.

In addition, during extrusion, amylose molecules interact with whey proteins to produce insoluble polymers, and increased polymer interactions will reduce the water-holding capacity of starch and protein, thereby reducing the amount of soluble protein [90]. Chen et al. [98] reported that increasing the proportion of soy protein isolate from 10% to 40% in a soy protein–corn starch mixture resulted in a decrease in the expansion rate of the mixture after extrusion. de Mesa et al. [99] also showed that when 20% soy protein was added, the integrity of starch particles in corn flour was damaged, resulting in a decrease in the expansion rate of the extrudates. However, several studies of extrusion-treated soy protein–starch products have shown the opposite results. Philipp et al. [100] found that the addition of pea-dissociated protein (PPI) had a great effect on the physical properties of rice starch extrudates, such as expansibility, density, and texture. When the amount of PPI is 10%, the expansion of rice starch can be promoted. The studies above show that extrusion processing can change the physicochemical properties of starch/protein and improve protein quality and protein and starch digestibility while providing some desirable functional properties.

### 5.2. Starch and Lipids

**Figure 1:**
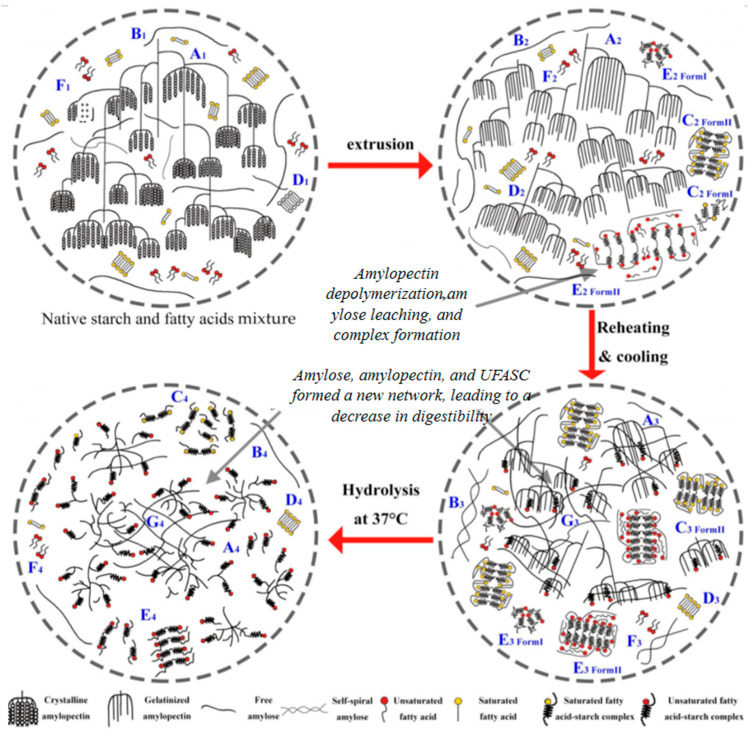
Schematic diagram of the fatty acid–starch complex formed by the extrusion, reheating, cooling, and hydrolysis process. Adapted from [102]. All rights reserved. Rights and permission from Elsevier.

Lipids are typically found in small quantities in food formulations during extrusion, as they reduce the friction necessary for transferring mechanical and thermal energy. Lipids can act as plasticizers to enhance the viscosity of specific products. Several studies have documented that extrusion leads to a reduction in lipid content [101]. The decrease in fat content may be attributed to the melting state of the material within the extrusion chamber during the process. This causes the double helix structure of starch molecules to loosen, allowing free fat to partially embed between and within starch helices. Consequently, there is a reduction in free fat content [91], as depicted in Figure 1. Furthermore, it has been demonstrated that lipids primarily form complexes with the amylose present in starch. Therefore, previous studies have proposed a squeeze-debranching strategy aimed at effectively gelatinizing and degrading starch through initial extrusion and subsequent enzymatic branching of amylopectin during a second extrusion step to improve the amylose content [92].

In the extrusion process of starch, fat will break down into small molecular fatty acids, which form a starch–fat complex, also known as RS5, when embedded in the helical hydrophobic cavity of starch molecules [103]. Therefore, the content of RS in the extruded material was positively correlated with the content of the complex, which may be because the extruded starch–fat complex is a kind of RS. Meng et al. [104] used the ultra-high pressure homogenization method to prepare a starch–fat complex and found that homogenization treatment significantly increased the content of RS in the material. In addition to the role of lipids as plasticizers to reduce friction and increase product viscosity during extrusion, the influence of extrusion parameters on the acquisition of starch–lipid complexes (especially RS5 resistant starch) is also critical. These parameters include extrusion temperature, screw speed, etc., which work together in the extrusion chamber and affect the interaction between starch and lipid and the formation of a complex. Firstly, extrusion temperature is one of the key factors affecting the formation of a starch–lipid complex. As the temperature increases, the double helix structure of starch molecules becomes more flexible and more easily interacts with lipid molecules. A suitable high temperature can promote the infiltration of lipid molecules into the starch spiral structure and form a stable starch–lipid complex [101]. Secondly, the screw speed indirectly regulates the mixing degree and complex formation of starch and lipid by influencing the residence time and shear force of the material in the extrusion chamber. The higher screw speed increases the shear force on the material, which is conducive to the more uniform dispersion of lipid molecules in the starch matrix and promotes the formation of the complex [92].

### 5.3. Starch and Non-Starch Polysaccharides

**Figure 2:**
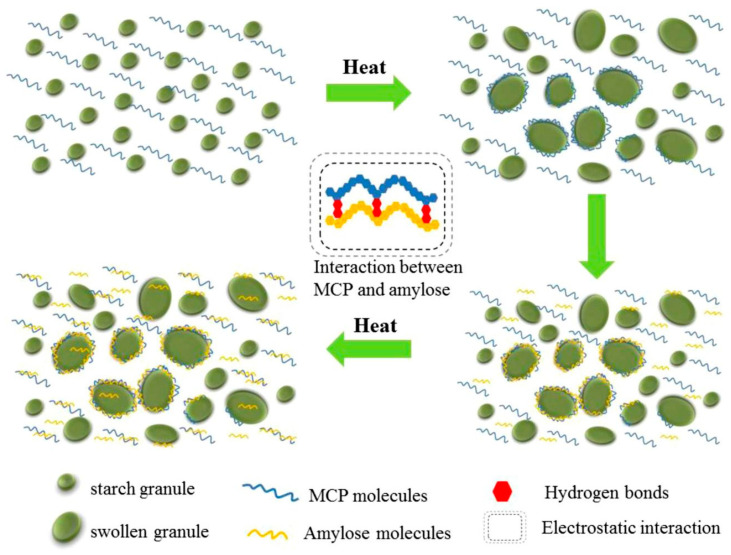
Interaction between rice starch and polysaccharide of rhizome. Adapted from [108]. All rights reserved. Rights and permission from Elsevier.

Non-starch polysaccharides (NSPs) are complex polysaccharides other than starch, such as dietary fiber, xanthan gum, and chitosan. They have good functional and processing characteristics [105,106]. In the extrusion process, NSPs can also interact with starch particles through hydroxyl groups to promote the formation of a more orderly network structure and further increase the binding effect on water, thus enhancing the stability of the composite system, as shown in Figure 2. He et al. [107] used the screw extrusion method to prepare rice starch containing different contents of konjac glucomannan. With the increase in konjac glucomannan concentration, the hydrogen bond force formed between konjac glucomannan and rice starch molecules under screw extrusion is strengthened, which increases the long- and short-range order degree and single and double helix structure of rice starch, showing a highly ordered structure.

Second, in the non-thermal processing mode, through extrusion and other mechanical treatments, in the NSP–starch interaction, starch particles become smaller and are broken from a high-polymer to a low-molecular structure so that the apparent viscosity of the complex increases [109]. For example, Zeng [110] found that after extrusion, xanthan gum has a higher viscosity and good rheology, and the viscosity of the xanthan gum–octenyl succinic anhydrate starch complex is enhanced. In contrast, starch has high stability at the beginning of the shear, but with the increase in the shear rate, its stability decreases, resulting in the decrease of apparent viscosity. However, in the case of thermal processing, heating breaks the hydrogen bond of the complex, and the viscosity of the starch decreases. In addition, NSPs can be attached to the starch surface by extrusion and other mechanical processing methods to form a physical barrier to resist the damage of mechanical shear force during starch processing. This physical barrier also blocks digestive enzymes, thereby inhibiting starch digestion and regulating GI [111]. For example, when rice starch–konjac glucomannan was assisted in the screw extrusion to form a complex, the content of RDS decreased significantly, from 48.06% to 36.47%, after the konjac glucomannan was added. Moreover, the SDS and RS contents increased from 16.81% to 22.39% and 35.13% to 41.14%, respectively [93].

### 5.4. Starch and Polyphenol

Phenolic compounds have attracted extensive attention due to their antioxidant and anti-inflammatory activities and are important new dietary functional supplements. However, extrusion has short-time continuous processing characteristics (the reaction time is usually 1–5 min). Compared with traditional curing methods, extrusion can not only achieve the rapid gelatinization and degradation of starch-based materials, such as grains, but also moderately retain endogenous phenols and other active components in materials. Ti et al. [94] studied the phytochemical characteristics and antioxidant activities of unprocessed and extruded black rice. The data showed that the content of free phenol, bound phenol, and total phenol decreased by 79.5%, 24.5%, and 71.2%, respectively. Zhang et al. [112] found that extracted rice samples still contained seven original phenolic acids, namely, ferulic acid, coumaric acid, p-coumaric acid, chlorogenic acid, gallic acid, caffeic acid, and syringic acid. The results show that extrusion did not change the types of polyphenols, and the free and bound phenols of milled rice and brown rice decreased by 53.7% and 40.1%, respectively. However, the total phenolic content of rice bran increased by 7.3% after extrusion. This experimental result was mainly attributed to the fact that the starch content of rice grain was higher than that of rice bran, and starch gelatinization formed a complex during extrusion that combined some polyphenols, resulting in the decrease in the free phenolic content. However, an extreme extrusion reaction microenvironment (high temperature, high pressure, high shear) may still cause the degradation and oxidation of phenols, reducing the phenolic content and antioxidant properties [113]. Therefore, the stability effect of the process depends largely on the selection of appropriate process parameters. In order to optimize the retention of phenolic substances and the formation of a starch–phenol complex in the extrusion process, it is very important to select the appropriate process parameters. These parameters include extrusion temperature, screw speed, material moisture content, etc., which jointly affect the physical state and chemical reaction rate of the material in the extrusion chamber. Through the precise control of these parameters, the gelatinization degree of starch and the infiltration, binding, and degradation process of phenolic substances can be controlled so as to realize effective retention of phenolic substances and the stable formation of a starch–phenol complex.

It has been reported that an endogenous phenol loss can be significantly reduced when amylase is properly introduced into the extrusion system [114]. The introduction of exogenous enzymes in enzymatic extrusion can loosen the overall structure of starch and break the molecular chain, thus producing starch degradation products with a large amount of exposure to hydroxyl groups [115,116]. This structural change may provide more sites for the conjugation of exogenous phenol and starch and better conditions for the molecular interaction and binding between starch and exogenous phenol in the system. Consequently, it has a high application prospect in starch–phenol complex processing. At present, relatively few studies have considered the use of enzymatic extrusion technology for starch–phenolic compound processing. Chen et al. [117] prepared starch-to-resveratrol particles using enzymatic extrusion technology and found that the addition of α-amylase in the extrusion process could significantly improve the release rate and photostability of resveratrol from the particles.

## 6. Conclusions

The extrusion process directly impacts the physical properties of starch, regulates the quality of the final product, and holds significant potential in the production of starch and starch-based food. The influence of extrusion on grain structure, gelatinization, and texture properties of starch is substantial. Altering the extrusion parameters can regulate the degree of gelatinization in starch, enhance interactions between starch and other food components (such as lipids and phenols), and affect both the microstructure and macroscopic properties of food. Consequently, this directly modifies physicochemical parameters in both starch itself and its derived products. These changes not only impact taste, texture, and stability but also exert crucial influence on nutritional value and digestibility. Despite some progress made in current research endeavors, most studies still focus on treating individual components within starch rather than fully elucidating complex interactions among matrix components, like protein and lipids, during actual processing that comprehensively affect food structure and performance. Therefore, future research should further explore how extrusion techniques impact grain-based starchy materials, particularly within emerging applications, to gain deeper insights into mechanisms governing the interaction between starches and other food ingredients. Simultaneously, efforts should be directed towards developing novel extrusion processing technologies that more effectively control alterations in a raw material’s composition while optimizing characteristics specific to starchy foods, thereby improving product quality, as well as nutritional value, to meet consumer demands for healthy yet delicious, convenient foods.

## Figures and Tables

**Table 1:** Effect of extrusion processing technology on physicochemical properties of starch.

Physicochemical Properties of Starch	Mechanism	Main Result	Application	References
Starch gelatinization	Weakening of hydrogen bonds	The starch particles quickly absorbed water and swelled, and the gelatinization temperature of starch decreased due to the effects of heat, shear force, and pressure.	Expanded product	[46,48,49]
	Breakage of starch particle structure			
	Extraction of amylose			
Starch retrogradation	Reorganization of hydrogen bonds between amylose and amylopectin molecules	The molecular chain of starch modified by extrusion interacted with the water ion, thus delaying the retrogradation of starch.	Fast-food product	[50,51,52]
	Cross-linking of starch molecules			
	Formation of local crystalline regions			
Starch rheology	Breaking of glycoside bond inside starch molecule	Starch viscosity reduction	Instant powder	[53,54,55]
Starch digestion	Damage to the integrity of starch particles	The degradation of starch molecules led to the formation of surface cracks, pits, and holes.	Easy-to-digest food for the elderly	[56]
	Increased contact area between starch and amylase			
	Breakdown of covalent hydrogen bond and crystal structure of starch particles	The content of resistant starch increased, and the digestibility of starch decreased.	Low-glycemic-index, starchy foods	[57,58,59]
	Rearrangement of starch molecules			
	Combination of starch with other substances (e.g., fatty acids, proteins, polyphenols)			

**Table 2:** Effect of extrusion processing technology on the interaction of various components of starch-based food.

Food Component	Primary Force	Structural Change	Result	References
Protein	Covalent bond Electrostatic interaction Hydrogen bond van der Waals Hydrophobic interactions	Hydrolysis of proteins	Improved protein quality Improvement of protein digestibility	[71,85,86]
		Unfolding and rearrangement of denatured structures		
		Cross-linking and polymerization between proteins and starches		
Lipids	Hydrogen bond van der Waals	Decomposition of lipids	Reduced free fat content The content of resistant starch increased	[73,90]
		Embedded in the spiral hydrophobic chamber of the starch molecule		
		Formation of a starch–fat complex		
Non-starch polysaccharides	Hydrogen bond van der Waals Hydrophobic interactions	Interacts with starch particles through hydroxyl groups	The apparent viscosity of NSP–starch complexes increases Inhibited starch digestion Regulated glycemic index	[91,92]
		Promotes the formation of a more orderly network structure		
		Increased binding to water		
		Enhances the stability of the composite system		
		Attaches to the starch surface		
		Forms a physical barrier		
		Resistance to mechanical shear damage during starch processing		
Polyphenol	Hydrogen bond Electrostatic interaction Hydrophobic interactions	Loosening of the overall structure of starch	Decrease in free phenol content Retention of active nutrients	[93,94]
		Breaking of molecular chains		
		Production of starch degradation products with large exposure to hydroxyl groups		
		Provides more sites for the coupling of exogenous phenols to starch		

## Data Availability

No new data were created or analyzed in this study. Data sharing is not applicable to this article.
